# Cigarette smoking and air pollution exposure and their effects on cardiovascular diseases

**DOI:** 10.3389/fpubh.2023.967047

**Published:** 2023-11-17

**Authors:** Manthar Ali Mallah, Tahmina Soomro, Mukhtiar Ali, Sobia Noreen, Nafeesa Khatoon, Akriti Kafle, Feifei Feng, Wei Wang, Muhammad Naveed, Qiao Zhang

**Affiliations:** ^1^College of Public Health, Zhengzhou University, Zhengzhou, China; ^2^Department of Sociology, Shah Abdul Latif University, Khairpur, Pakistan; ^3^Department of Chemical Engineering, Quaid-e-Awam University of Engineering, Science and Technology, Nawabshah, Sindh, Pakistan; ^4^Department of Pharmaceutics Technology, Institute of Pharmacy, University of Innsbruck, Insbruck, Austria; ^5^School of Nursing, Zhengzhou University, Zhengzhou, China; ^6^Department of Physiology and Pharmacology, College of Medicine and Life Sciences, University of Toledo, Toledo, OH, United States

**Keywords:** cardiovascular diseases, air pollution, air particulate matter, cigarette smoking, inflammation

## Abstract

Cardiovascular disease (CVD) has no socioeconomic, topographical, or sex limitations as reported by the World Health Organization (WHO). The significant drivers of CVD are cardio-metabolic, behavioral, environmental, and social risk factors. However, some significant risk factors for CVD (e.g., a pitiable diet, tobacco smoking, and a lack of physical activities), have also been linked to an elevated risk of cardiovascular disease. Lifestyles and environmental factors are known key variables in cardiovascular disease. The familiarity with smoke goes along with the contact with the environment: air pollution is considered a source of toxins that contribute to the CVD burden. The incidence of myocardial infarction increases in males and females and may lead to fatal coronary artery disease, as confirmed by epidemiological studies. Lipid modification, inflammation, and vasomotor dysfunction are integral components of atherosclerosis development and advancement. These aspects are essential for the identification of atherosclerosis in clinical investigations. This article aims to show the findings on the influence of CVD on the health of individuals and human populations, as well as possible pathology and their involvement in smoking-related cardiovascular diseases. This review also explains lifestyle and environmental factors that are known to contribute to CVD, with indications suggesting an affiliation between cigarette smoking, air pollution, and CVD.

## Graphical abstract

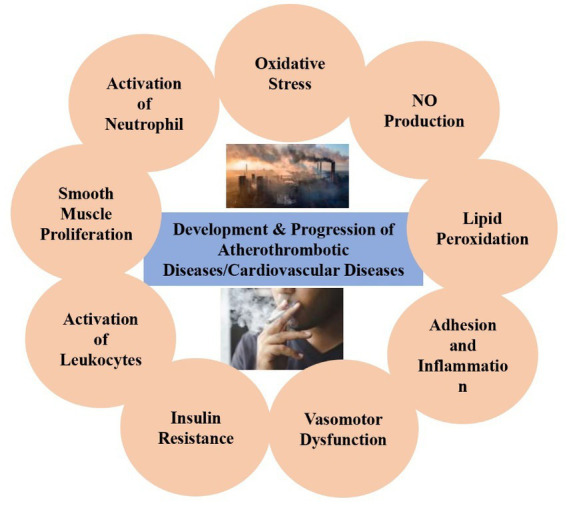

## 1. Introduction

Air contamination or pollution is the foremost environmental risk factor, accounting for roughly 1/9th of all fatalities worldwide according to the World Health Organization (WHO) (1). In the air, the PM_2.5_ (particulate matter with a diameter of 2.5 μm or less) has a strong link to vascular effects that contribute to myocardial infarction (MI), ischemia, strokes, and additional cardiovascular diseases (CVDs) (2–4). The influence of air quality on cardiovascular health is comparable to pulmonary problems (3, 5), as found the lung cancer (6). Epidemiological studies describe that contact with particulate substances in the atmosphere may increase CVDs (7). According to epidemiological studies, cigarette smoking is the leading cause of fetal coronary artery disease (CAD) and myocardial infarction (MI) (8). Studies show that passive smokers having excessive exposure to the tobacco smoke environment (1/100th) have approximately 30% more risk factors for CAD as compared to active smokers (80%) (9). Furthermore, passive smoking (ambient tobacco exposure) is connected to a 30% rise in the risk of coronary artery disease, comparable to 80% rise in active smokers (10, 11). Thus, while there is strong evidence relating cigarette smoke exposure to cardiovascular disease, the elements of cigarette smoke and the pathways that underpin such a relationship are still unknown. The main aim of the current study is to demonstrate the findings on the effect of CVD on the health of individuals and human populations, possible pathology, and their involvement in smoking-related cardiovascular diseases. This review also explains environmental factors and lifestyle that are known to contribute to CVD, with evidence indicating a link between air pollution, cardiovascular disease, and cigarette smoking.

## 2. The burden of cardiovascular diseases

A significant health concern worldwide is CVD, responsible for 30% of all deaths (12). CVD was accountable for 17.5 million of the 58 million deaths from all causes globally in 2005, three times the number of fatalities caused by infectious illnesses like tuberculosis (TB), HIV/AIDS, and malaria (13–15). In 2030, non-communicable diseases are expected to be responsible for more than three-quarters of all fatalities, and CVD fatalities will increase to 23.4 million, almost 37% higher than the 2004 rates. In addition, cerebrovascular disease (stroke) and ischemic heart disease (IHD), both components of CVD, are expected to be the leading cause of death worldwide in 2030 (14).

CVD has no boundaries in terms of geography, socioeconomic status, or gender, according to the WHO. CVD is expected to be the most significant cause of mortality in emerging nations, indicating that it is not limited to the most developed. Nearly 80% of fatalities from CVDs occur in low- and middle-income nations (15). Lower socioeconomic groups in developed countries have a higher frequency of risk factors, a greater incidence of illness, and a higher death rate. The higher incidence of the disease will transfer to lower socioeconomic groups as the CVD epidemic evolves in emerging nations (15). It is reported that CVD is also the major cause of death in women around the world (16).

CVD is a main source of early death and growing healthcare costs (13, 14). The main drivers of CVD are cardio-metabolic, behavioral, environmental, and social risk factors. However, some important risk factors for CVD (such as tobacco smoking, poor diet, and lack of physical exercise), and environmental pollution have also been linked to an elevated risk of cardiovascular disease. Identifying and understanding the effects of such toxins will improve our understanding of the mechanisms of disease progression, which should lead to improved interventions to reduce CVD in human populations. Thus, CVD is a public health issue that demands a global prevention method. Because of a lack of financial resources and professionals with expertise in CVD prevention and management, its influence is highest in developing countries. Global prevention actions need to consider phases of development in nations and areas.

## 3. Lifestyle and environmental factors contribute to cardiovascular disease

Lifestyles and environmental factors are known key variables in cardiovascular disease. Accumulating evidence indicates that exposure to toxins and chemicals may raise CVD risk. Exposure to tobacco smoke and the environment: air contamination is considered the source of toxins that contribute to the CVD burden.

### 3.1. Cigarette smoke exposure and its effect on cardiovascular physiology

**Figure 1:**
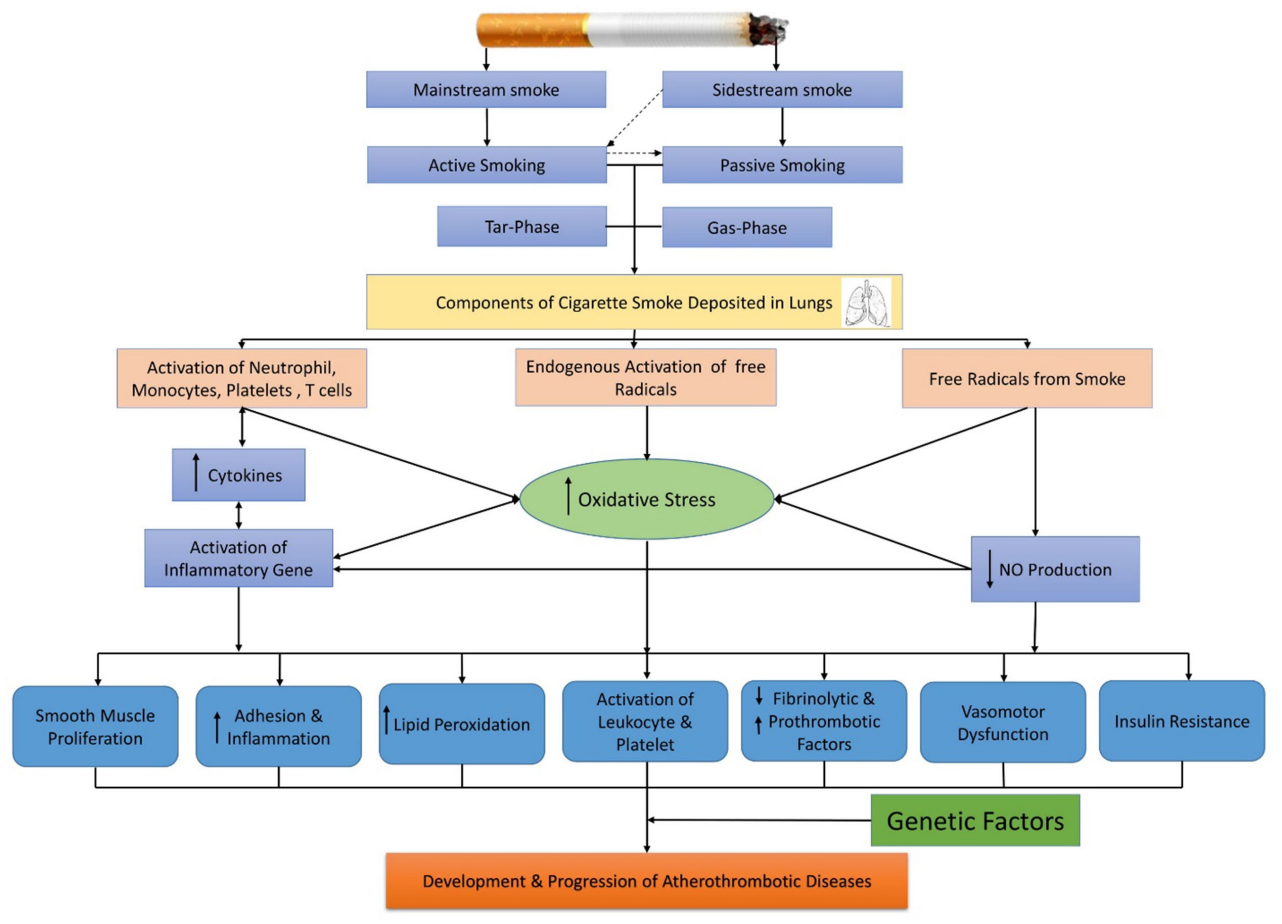
Cigarette smoking exposure and its mechanism for cardiovascular dysfunction.

Epidemiological research suggested that cigarette smoking is the foremost reason for fatal coronary artery ailment (CAD) and MI (8). Compared to non-smokers, low-tar cigarettes and smokeless tobacco have been linked to an increased risk of cardiovascular events (9). Studies show that passive smokers having excessive exposure to tobacco smoke environment (1/100th) has approximately 30% risk factors of CAD as compared to active smokers (80%) (9). Therefore, evidence is present that connects the consumption of cigarettes to several CVDs. However, the same ingredients of cigarette smoke and the pathways accountable for this relationship have not been openly articulated. Figure 1 shows some recent clinical and experimental findings that are responsible for the disease pathology of smoking-related.

#### 3.1.1. Chemical and physical characteristics of cigarette burn

The constituents of cigarette smoke may be classified into two stages: tar and gas. The substance, the tar or particle phase, contains >10^17^ free radicals/puff that are generated from the ash of the tobacco (stream of smoke) and passed by the cigarette filter (Cambridge-fiber filter) that contains all of the suspended particulate matter (99.9%) having a particle size range > 0.1 μm (17). On the other hand, the gas phase is the substance that contains >10^15^ free radicals/puff that passes through the filter and remains active for a shorter time than the tar phase free radical (17, 18).

Mainstream smoke is known as cigarette smoke, which is produced from tobacco through an active smoker’s mouth. Side-stream cigarette smoke is made from the burning end of a cigarette. 8% of tar and 92% of gaseous elements are composed of mainstream cigarette smoke (17). Environmental tobacco smoke is primarily made up of 85 percent side-stream smoke and a small percentage of aspirated mainstream smoke from consumers (19). A rise in the intensity of the noxious gaseous constituent is found in side-stream cigarette smoke compared to mainstream cigarette smoke. Nicotine, which is a component of the tar phase, is the most dangerous of all the identified elements of cigarette smoke (20).

#### 3.1.2. Smoking cigarettes and atherosclerosis

Cigarette smoking (CS) causes a range of therapeutic atherosclerotic syndromes, such as acute coronary syndromes, stable angina, stroke, and premature mortality. Atherosclerosis of the aortic and peripheral arteries is also increasing, resulting in claudication and gastrointestinal aortic aneurysms regularly (21).

The link between CS and atherosclerosis has been studied using a variety of clinical imaging techniques. Initial research attributed the pack smoking period to the magnitude of angiographic atherosclerosis (22). Other techniques precisely predict the atherosclerotic changes related to cigarette smoke contact. Angiography is an inadequate parameter of the quantity or development of atherosclerosis. As evaluated by transesophageal echocardiography (ECG), thoracic aortic atherosclerosis (TAA) was enhanced in cigarette smokers (23). Moreover, it has also been demonstrated that a continuous rise in intimal-medial carotid artery thickness, as measured by carotid ultrasonography, is caused by both active and passive smoking (24). Lipid modification, inflammation, and vasomotor dysfunction are integral components of atherosclerosis development and progression. These constituents appear as the superficial clinicopathological and structural indicators of atherosclerotic (21). The present information about the impact of CS on these constituents of atherogenesis is discussed further in the following sections.

##### 3.1.2.1. Alteration of the lipid profile due to cigarette exposure

Smoking cigarettes may encourage atherosclerosis, partly, due to its impacts on lipid profiles. Smokers with a long history of smoking have slightly higher serum cholesterol, triglycerides (TG), and low-density lipoprotein (LDL). The distinguishing feature in such analyses is the high-density lipoprotein (HDL) exclusively found in smokers (25). However, the mechanical details are not adequately explained, and it is also uncertain how important the dietary differences between smokers and nonsmokers. Insulin resistance has recently been linked to HDL/triglyceride changes. Insulin resistance, in particular, has been identified as a possible key link between CVD and CS (26).

Furthermore, the oxidative state of LDL increases with CS. Such an increase in oxidation state profoundly alters the circulating metabolites of lipids and autoantibodies responsible for LDL oxidation (27). It was found that overexposure to cystathionine γ-lyase (CSE) results in the modification of LDL. The macrophages preferred this modified LDL and consumed it when forming foam cells in culture (28). Similarly, the exposure of the gas phase of cigarettes to human plasma cells results in the alteration of LDL in plasma (29).

Additionally, compared to HUVECs not available to non-smokers, HUVECs increasingly derived from smokers significantly elevated the oxidative alteration of LDL (30). The plasma activity of paraoxonase, an enzyme that inhibits LDL oxidation, may decrease with exposure to cigarette smoke.

##### 3.1.2.2. Inflammation due to cigarette exposure

The inflammatory reaction is an integral part of atherosclerosis development and progression. Several studies have observed that CS induces the peripheral blood leukocyte count to rise by around 20–25% (31). CS is associated with greater levels of inflammatory markers such as interleukin (IL)-6, C-reactive protein (CRP), and tumor necrosis factor (TNF)-αin smokers in both sexes (32, 33).

On the surface of endothelial cells, the activation of leukocytes is characterized by atherosclerosis. Numerous proinflammatory cytokines have advanced, increasing leukocyte-endothelial cell involvement and therefore leukocyte recruitment. Smokers have higher amounts of the intercellular adhesion molecules ICM-1, E-selectin, and vascular cell adhesion molecule (VCAM)-1 (34, 35). CS also induces the discharge of pro-atherogenic molecules, contributing to the alteration of cell–cell communications. CS treatment was linked to an increase in observation rates (70–90%) among HUVECs and human monocytes in culture due to the augmented initiation of adhesion molecules on the surfaces of both HUVECs and monocytes (32, 33). CSE boosted the rate of vascular endothelial cells relocation of monocyte-like cells along a HUVEC surface by 200%. The integrin CD 11b/CD 18 was greater in smokers’ monocytes, which increased monocyte adherence to HUVECs in culture. Likewise, Adams et al. discovered a significant growth in adhesion between HUVECs and human monocytes after exposing them to smokers’ serum, which was linked to enhanced ICAM-1 on HUVECs (32, 33). Thus, CS adds fuel to the fire of inflammation at the vessel wall and in the blood.

##### 3.1.2.3. Vasomotor dysfunction due to cigarette exposure

One of the initial atherosclerotic modifications in a vessel is vasodilatory function impairment. Several kinds of research have shown in animal and human models that cigarette smoke consumption, both passive and active, is linked to a reduction in its vasodilatory role. Smoking cigarettes damages endothelium-dependent vasodilation (EDV) in the brachial and coronary arteries, as well as other macro- and microvascular beds in humans (36, 37).

The endothelium’s vasodilatory activity is principally mediated by a free radical, which is nitric oxide (NO) (38). Utilizing isolated components like nicotine or cigarette smoke extract (CSE), it has been discovered that CS inhibits NO production *in vitro* (21, 39). Because the metabolic fate of several documented and unidentified cigarette smoke components in human is uncertain, a suitable *in vitro* CS exposure model must be developed. Our team has fostered endothelium cells with smokers’ sera to generate a more physiologic *in vitro* model. Barua et al., using this model (40, 41) found that by modifying the endothelium’s NO synthase enzyme’s expression and activity, exposure to smokers’ sera reduced the availability of NO from human coronary artery endothelial cells (HCAEC) and HUVECs. In HUVECs exposed to the same individuals’ serum in culture, there was a significant link between circulation brachial artery EDV and NO availability (40). Similarly, other studies using N^G^ monomethyl-L-arginine (L-NMMA) *in vivo* infusion have ultimately found that the reduced smoking-related EDV was responsible for reduced NO production (42).

NO is a vaso-regulatory molecule that also helps in regulating thrombosis, platelet activation, leukocyte adhesion, and inflammation (38). Therefore, modifications in NO production may have both primary and secondary impacts on the development and progression of atherosclerosis as well as thrombotic events.

#### 3.1.3. Cigarette smoking and thrombosis

An increased frequency of acute MI is associated with cigarette smoking. In the first 1–3 years after giving up, this risk is greatly reduced, and after 5 years, the risk approaches that of an ex-smoker (43, 44). The latest results suggest that thrombotic incidents of smoking abstinence are immediately reduced. Preliminary oral presentation research recorded that over 6 months in Helena, Montana, a citywide smoking ban in public places minimized the frequency 60% of severe MI. In men, CS also raises the hazard of plaque rupture as well as severe thrombosis of a lipid-rich, thin-capped Roma; plaque erosion with superimposed thrombosis was the primary mechanism in smokers (45). Acute cigarette smoke exposure can reduce coronary blood flow and increase vascular coronary artery resistance. The risk factor for coronary vasospasm could also be smoking (46).

It has been consistently shown that cigarette smoke exposure’s prothrombotic effects induce modifications in fibrinolytic factors, antithrombotic/prothrombotic factors, and platelet function. The current information on these effects is discussed in the following sections.

#### 3.1.4. Fibrinolysis modification due to cigarette exposure

A substantial decrease in the t-PA/PAI molar ratio and significant reductions in both baseline and substance-P-stimulated t-PA discharge were observed in HUVECs exposed to chronic serum smokers (47). After pharmacologic activation, samples isolated from coronary and brachial arteries revealed lower activity in smokers and plasma t-PA antigen (48).

As a result, CS is linked to malfunctioning thrombo-hemostatic systems that aid in the onset and dissemination of thrombus formation.

##### 3.1.4.1. Platelet dysfunction due to cigarette smoke

An enhanced stimulatory and spontaneous aggregation of platelets isolated from smokers was observed (49). Platelets evaluated from non-smokers showed hyperaggregability after exposure to smokers’ serum (50). CS can reduce the accessibility of NO derived from platelets and decrease the sensitivity of platelets to exogenous NO, resulting in increased activation and permeability (51).

##### 3.1.4.2. Modification of antithrombotic/prothrombotic factors due to cigarette exposure

There are higher fibrinogen levels in active smokers associated with the number of cigarettes consumed. Ex-smokers have a comparable amount of fibrinogen as non-smokers. Increases in TF pathway inhibitor (TFPI)-1 and tissue factor (TF) have also been documented, along with an increase in thrombotic capability. HUVECs uncovered in chronic smokers’ serum showed a significant drop in TFPI-1 and a moderately noteworthy but quasi-rising TF level (47). Increased TF immunoreactivity and action were seen in the atherosclerotic plaques of apoE mice exposed to half of a non-filtered study cigarette 5 days per week for 8 weeks (52). An increase in circulatory TF action has been observed in human plasma after consuming two cigarettes within 2 h (53). In comparison, the prothrombotic process related to smoke exposure is potentiated by elevated hematocrits, blood viscosity, red blood cell counts, and a continuing inflammatory mechanism (31).

#### 3.1.5. Factors and mechanisms of vascular dysfunction caused by cigarette exposure

In cigarette smoke, there are about 4,000 identified components, although only a few have been investigated in isolation. One such variable is carbon monoxide (CO), yet its implications on atherothrombotic ailment have been ambiguous. Previous research proposed that CO might be accountable for cardiovascular changes associated with smoking (54). However, more recent findings show that cigarette smoke’s CO is an unusual source of thrombus or atherosclerosis (39, 55).

The most studied factor is the potential presence of nicotine in cigarette smoke. Although nicotine has a substantial impact on alterations in heart frequency, blood stress, and cardiac output caused by smoking, its involvement in atherothrombotic illness related to CS is still debated (56). Exposure to nicotine alone was stated to cause no variation, increase, or decrease in the supply of NO or EDV (57). While high dosages of nicotine support atherogenic improvements in several models, most existing research shows that nicotine has a slight impact on the onset or spread of atherosclerosis at concentrations comparable to those of a smoker’s blood level (31, 57). Similarly, in the context of smoking, nicotine appears to have no effect on thrombo-hemostatic features such as platelets or t-PA, PAI-1, or fibrinogen (31, 58). As previously mentioned, nicotine is a recognized addictive constituent of cigarette smoke, addiction to it is likely to spread to other, riskier components.

In the development of atherosclerosis, free radical-mediated oxidative stress is emerging as a critical phase (59, 60). Free radicals could arise in a CS setting from: (1) the gaseous phase of the CS; (2) migrating or *in situ*-stimulated neutrophils and macrophages; and (3) natural means producing reactive oxygen species (ROS) like the mitochondrial electron transport chain (ETC), derived endothelial nitric-oxide synthase (eNOS) and xanthine oxidase (61–63). Cellular oxidative stress increases due to interaction among free radicals like superoxide and NO, which creates peroxynitrite (64). Enhanced oxidative stress shifts cellular stability into a proatherogenic and prothrombotic state without the protective influence of NO (65, 66). A number of the abnormalities mentioned above, such as proinflammatory consequences on the vessel wall, prothrombotic effects such as augmented platelet reactivity, decreased endogenous fibrinolysis, lipid peroxidation (LPO), and endothelial dysfunction, can all be explained by elevated oxidative stress (67–69). Additionally, CS’s proatherogenic, prothrombotic, and proinflammatory properties have been found to augment or reverse antioxidants or treatments that reduce oxidative stress or increase NO availability (41, 70).

#### 3.1.6. Smoking’s non-linear dosage effect on cardiovascular dysfunction

While the link between CS and CVD risk has been documented, the query of whether or not there is a direct dose effect remains unanswered. A dose-dependent, solid relationship between CVD risk and the total amount of cigarettes consumed or the number of exposure years in a pack has not been recognized in many recent major epidemiologic studies, indicating a tendency toward more cardiovascular events in heavier, active smokers (71). Following that, both heavy and light active smokers found consistent reductions in EDV, and NO production anomalies in the brachial artery (72). Similarly, specific atherothrombotic indicators, a lower EDV and even increased platelet activation with passive smoking were comparable to active smoking (71). The mentioned findings recommend that tiny doses of noxious tobacco smoke elements can cause the major biochemical and cellular progressions to become saturated on cardiovascular function, resulting a non-linear dose response. The particular appliances they comprise need to be investigated further.

### 3.2. Environmental exposures: air pollution

Notably, while airborne chemicals can potentiate CVD risk factors, noise exposure, for example, are non-chemical environmental variables; temperature, occupational dangers, socioeconomic factors, electromagnetic fields, mental/psychosocial stress, agricultural practices, built environments, and changes in the artificial climate and ecosystem can all interact with air pollution, potentially amplifying the link between the two (73).

#### 3.2.1. Chemistry, composition, and sources of air pollution

Polluted air is a constantly changing combination of particle and gaseous phase components that vary in location and time (3, 74, 75). The effects of air pollution on the environment are a utility of chemistry, and simplistic arrangements give an imperfect image centered on single contaminants, mass, or size. The atmospheric PM is a complex mixture of particulates and liquid droplets with a broad range of chemical composition (76). PM is categorized based on particle size, with coarse particles (diameter between 2.5 and 10 μm) primarily originating from construction and demolition, mining, agriculture, tire destruction and road dust. Fine particles (diameter ≤ 2.5 μm; PM_2.5_) and ultrafine particles (also known as nanoparticles; diameter ≤ 0.1 μm) come from both natural and anthropogenic sources. For convenience, the proportion of air emission particulate matter (PM) is generally defined by aerodynamic thickness: <10 μm [thoracic particles (PM_10_)], <2.5 μm [fine particles (PM_2.5_)], <0.1 μm (ultrafine particles), and between 2.5 and 10 μm [coarse (PM_2.5–10_)]. PM is measured per cubic meter (μg/m^3^) by the particles (mass) produced. In metropolitan areas, however, gasses/air, or vapor-phase chemicals, such as volatile organic carbons, make up approximately 90% of the contaminants in the air we breathe (3). In addition to various organic and inorganic acids, semi-volatile organic compounds, and volatile organic compounds that are present in both the gas phase and particle phase, the most predominant secondary contaminant is ozone. While a growing body of studies has shown the danger of coarse PM and possibly ultrafine PM (77–79), the vast majority of confirmation disproves PM_2.5_ as the main air contaminant causing the utmost damage to the well-being of the world’s inhabitants.

##### 3.2.1.1. Air adulteration from household

Often seen in low–middle-income countries, household air pollution levels can be an order of magnitude higher remarkable in the same geographic area than atmospheric outdoor levels due to gradients between indoors and outdoors (80). For example, mean indoor 24-h PM_10_ levels ranging from 200 to 2000 μg/m^3^ are relative. Peak exposures >30,000 μg/m^3^ have been recorded during cooking cycles offering lower biomass fuel combustion (80, 81). In comparison to a decade ago, the drop in household air pollution’s contribution to world morbidity and mortality is promising.

##### 3.2.1.2. Dust involvement from natural sources

Elements from natural phenomena such as explosions, volcanic eruptions, and desert sands have been shown to have negative health effects, and have recently been called to attention. Natural dust accounts for 18% of all early deaths caused by polluted air (82).

#### 3.2.2. Thresholds of air pollution and risk assessment

More than 90% of the world’s population is exposed to levels that are greater than the WHO’s air quality standards (AQG), which are <10 g/m^3^ annually and < 20 g/m^3^ daily. Air contamination regularly reaches 35 μg/m^3^ in many areas of Asia (70% of the population), and over 99% of residents are subjected to AQG levels that are higher than the average. During severe periods of ambient air pollution, PM_2.5_ concentrations can even surpass notable levels beyond 500–1,000 g/m^3^, values comparable to high levels of indoor air pollution or active smoking (80, 83). On the other hand, values regularly average < 12 μg/m^3^ in Canada and the USA. The present improvement in awareness of global air contamination exposure is not at all minor and includes air quality index data hourly from regulatory checking data networks from more than 9,000 locations in right 100 cities across 70 countries due to the drastic increase of ground-level air-monitoring data. Enhanced awareness in some cases, regulatory reforms have led to non-traditional measuring outlets, social media and independent stations (by means of personal level monitoring) and air evaluators are examples. The breadth and reach of customized tracking systems have also significantly increased, with several of these devices being available to offer unparalleled knowledge on personal exposure (84). Satellite-based techniques are applied to obtain indirect evaluations of ground-level pollutant concentrations using the visual characteristics of aerosol columns (aerosol optical depth) in satellite pictures, which form the basis of Global Burden of Disease (GBD) air quality estimations. The progress of an interactive contact-response arc that combines relative risk of PM_2.5_ effluence from various causes (household air pollution, ambient air pollution, tobacco smoking, and second-hand smoke) into a single curve on which risk levels are calculated addressed the need for credit risk estimates for nations with air pollution concentrations beyond those of the sample populations (85, 86). Remarkably, the entirety of the evidence suggests no lower concentration threshold at which population-level concentrations can be deemed healthy. Also, within the annual AQG of less than 10 μg per cubic meter, the little amount of PM_2.5_ goals faced by hundreds of millions of individuals pre suppose substantial public health risks and, especially, the CV events (79, 87–91). In addition, other air contaminants (e.g., ozone) could pose an individual risk of their own (90, 92). Compared to PM_2.5_, ozone displays a threshold impact from short-term exposure in the most recent estimates (91).

#### 3.2.3. Air pollution and disease burden-mediated morbidity and mortality

PM_2.5_ overwhelmingly influenced nations over the past century, for instance, India and China, due to ecological-economic changes; it influences almost all globally (73, 75, 83, 93–95). The most recent GBD estimates state that ambient PM2.5 caused 4.2 million fatalities and 103.1 million disability-adjusted life-years (DALYs) in 2015, which accounted for 7.6% of all global fatalities and 4.2 percent of all DALYs worldwide (73). Due to the use of solid fuels, household air pollution resulted in 85.6 million DALYs and 2.8 million fatalities in 2015. CV ailments accounted for more than half of the health risks (6, 73). Ozone exposure caused 4.1 million DALYs and 254,000 deaths from chronic obstructive pulmonary disease (COPD) in 2015 (73).

In a current report, it is stated that in the Medicare population (61 million US residents), short-term increases in PM_2.5_ and ozone were connected to daily death rates of 1.0105 (95%CI = 1.0095–1.0115) and 1.005 (95%CI = 1.0041–1.0061), respectively (91). Despite the lack of cause-specific mortality statistics, a large body of research suggests that CV factors account for more than half of all fatalities caused by air pollution. Furthermore, even though this study did not allow for testing the association of variables like income and smoking on the relationship, a subsequent analysis of the Medicare Current Beneficiary Survey found no such interaction (90). These findings are comparable with research from Canada and Europe that shows a roughly linear connection between PM_2.5_ and fatalities at PM_2.5_ g/m^3^ levels (87, 96, 97).

#### 3.2.4. Cardio-metabolic effects of air pollution

##### 3.2.4.1. Heart failure due to air pollution

A transient rise in gaseous components and PM (both PM_10_ and PM_2.5_) was associated with an increased risk of heart disease hospitalization or mortality, as shown by a systematic review and meta-analysis of 35 studies (98). Owing to heart letdown, the relative risk (RR) of hospitalization or death enlarged by 2.1% as a result of the 10 g/m^3^escalation in PM_2.5_ (RR = 1.021; 95%CI = 1.014–1.028). In a new Chinese revision of 26 towns with high PM_2.5_ concentration, an increase in PM_2.5_ at the inter quartile was linked to a 1.3% relative rise in heart failure hospitalizations (99).

##### 3.2.4.2. Cerebrovascular ailment due to air pollution

It was discovered that a 10 g per cubic meter increase in PM_2.5_ and PM_10_ concentrations was related to a 1 % increase in the risk of hospital admission with stroke and stroke fatalities, which has been conducted by SMA in 94 research work comprises of 28 countries (100). It seems that existence nearby the road and poverty are related to ischemic stroke and the incidence of stroke (101, 102). Despite the fact that the link was not statistically noteworthy in the primary evaluations, there was a connection between PM_2.5_ and mortality in the ESCAPE cohort study of subjects 60 years of age (hazard ratio (HR) = 1.40; 95% CI = 1.05–1.87 per 5 g/m^3^ increase in PM_2.5_), non-smokers (HR = 1.74; 95% CI = 1.06–2.88 per 5 g/m3 rise in PM_2.5_), as well as among contributors (79). Greater risk was mainly observed in participants over the age of 60 years, as well as in non-smokers, and was reliably found at PM_2.5_ < 25 μg/m^3^ meditations. With significant increases for longer time periods of PM_2.5_ contact of 35 and 83.0%/10 g/m^3^, correspondingly, the Women’s Health Initiative study in the United States revealed certain of the highest evaluations of stroke and demise from cerebrovascular sickness owing to PM_2.5_ (103).

##### 3.2.4.3. Myocardial infarction due to air pollution

The relationship between daily variations in MI and short-term increases in air pollution has been investigated in case crossover as well as time-series studies across the globe. A systematic review and meta-analysis of studies of short-term exposure to air pollution and MI found a relationship between the increased risk of MI and PM_2.5_, sulfur dioxide, nitrogen dioxide (NO_2_), and carbon monoxide (104). In the ESCAPE (Cohort Studies in Europe for Air Pollution Effects) study (*N* = 100,166 from different cohorts) a substantial 13% relative rise in nonfatal acute coronary events was found, with a 5 μg/m^3^ increase in long-term PM_2.5_ exposure (105). Exceptionally high risk can be found in a diseased person with a fundamental coronary blood vessel ailment. The greatest recent research on the relationship between concurrent-day PM_2.5_ and increase in acute coronary syndrome (ACS) on the same day comes from Utah (Intermountain Health Care, *N* = 16,314) (106). Only people with coronary artery angiographic disease were shown to have an increased risk of MI, which resulted in an escalation in ST fragment levels. Long-term survival resulting in ACS was similarly lowered as a result of long-term PM_2.5_ exposure (107, 108).

##### 3.2.4.4. Cardiovascular deaths due to air pollution

Exposure to PM_2.5_ for a time raised the comparative risk (CR) for severe MI by 2.5% per 10 μg cubic meters (RR = 1.025; 95%*CI* = 1.015–1.036) as reported in a number of investigations (104). Despite the low relative risks, short-term PM_2.5_ exposures are responsible for up to 5% of MI globally (attributable population fraction), even though hundreds of millions of people are constantly distressed (109). It has been suggested that monotonous, near-continuous acquaintance with air contamination promotes atherosclerosis and recurrent occurrences, considering that air impurity revelation happens across a lifetime (110). Certainly, longer-term exposures seem to pose amplified risks over several years (75, 103, 110–112). Ischemic heart disease fatalities increased considerably in two Canadian studies, despite PM_2.5_ being less than 9 μg per cubic meter on average (87, 89). In the National Organizations of Health–AARP cohort (*N* = 517,043) in the United States (US), similar findings were reported where long-standing exposure increased CV death by 10% (per 10 μg/m^3^) despite low PM_2.5_ levels (10–13 μg/m^3^) (113). There have been multiple studies conducted in China that suggest a rise in severe CV mortality when PM_2.5_ levels are high (114, 115). A meta-analysis of the research found that each 10 μg/m^3^ increase in PM_2.5_ was associated with an absolute 0.63 percent rise in the CV death rate, even though PM_2.5_ levels ranged from 39 to 177 μg/m^3^ (114). A few long-term cohort trials have performed elevated PM_2.5_ levels, indicating an improved effect of longer-period disclosure (116). However, at higher levels of air pollution (average PM_2.5_ levels of 43.7 μg/m^3^), the higher risk of morbidity and mortality persists which is stated in a recent study in China (117). The RR for CV fatality rose by an elevation of 9%/10 μg/m^3^, above the feature of the combined exposure-response arc. This recommends that additional longitude investigations are required to better illustrate the figure of the full dose–response arc at high levels of exposure to lethal and nonlethal CV events, given the impact on the burden of global public health.

#### 3.2.5. Cardio-metabolic risk mediated by air pollution

According to a large body of research, air pollution is increasingly implicated in the rise of cardio-metabolic threat factors such as insulin fighting and hypertension, according to a large body of studies.

##### 3.2.5.1. Hypertension mediated by air pollution

There have been at least four meta-analyses investigating the link between hypertension and air pollution in the past (118–121). For the following several days, increases in systolic and diastolic blood pressure of 1–3 mm Hg are reliably linked to an up surge in appropriate PM_2.5_ concentrations of 10 g per cubic meter. In several studies, longer-term exposures have been linked to a permanent rise in plasma force and an increased incidence or prevalence of hypertension. In meticulously planned, closely watched human experiments, a diversity of vascular alterations in reaction to air adulteration have been examined, and increases in blood pressure are frequently perceived (75, 111, 122–130).

According to published randomized controlled trials, short-term exposure causes higher plasma stresses or modifications in the vascular index. Robust interactions were observed in the Chinese megacity at exciting levels of the exposure-response association (131). Notably, unique lower air pollution techniques show accelerated blood pressure reduction, further supporting the significant influence of air fragment inhalation on plasma stress (75, 132). This body of research confirms increased blood pressure levels and morbidity associated with hypertension in the global burden estimates for air pollution.

##### 3.2.5.2. Insulin resistance/diabetes mediated by air pollution

Previous expert studies have reviewed the relationship between type 2 diabetes mellitus and insulin resistance (75, 111, 133, 134). The comparative risk for diabetes increased by 39% for every 10 g/m^3^ of PM_2.5_ according to a meta-analysis of cohort trials comprising a total of 2,371,907 people and 21,095 cases of type 2 diabetes mellitus (135). PM_2.5_ and NO_2_ increased the incidence of diabetes (HR = 1.10; 95%CI = 1.02–1.18 and HR = 1.08; 95%CI = 1.00–1.17 per 10 g/m^3^ increase in PM_2.5_ and NO_2_, respectively) stated in the utmost new meta-analysis (136).

#### 3.2.6. Cardiac arrhythmias mediated by air pollution

It has been shown that sudden air pollution exposure causes atrial fibrillation. The inhabitants’ attributable risk of atrial fibrillation increased by 0.89% (95%CI = 0.20–1.57) for every 10 g/m^3^ of PM_2.5_ in a meta-evaluation of four experimental research studies consisting of 461,441 people (137). The threat of ventricular arrhythmias has also been linked to air pollution exposure in the past, albeit the data is insufficient (74).

##### 3.2.6.1. Venous thromboembolism mediated by air pollution

The link between venous thromboembolism and exposure to contaminated ambient air is uncertain. Few types of research have indicated a relationship, although others have not (138, 139).

##### 3.2.6.2. Other non-communicable diseases mediated by air pollution

Other chronic cardio-metabolic syndrome-related diseases, including obesity (140), sleep-related respiratory disorders (141), chronic kidney disease (142), along with neurological diseases (e.g., dementia, sadness) (141), have been associated with air contamination, though further study is required.

#### 3.2.7. Mechanisms of air pollution-mediated cardio-metabolic disease

Although the explanation of the mechanisms underlying the systemic CV risk mediated by air pollution is still developing, there are six fundamental subordinates “effector” pathways: Endothelial barrier dysfunction, distinctive and adaptive immune constituents in inflammation, prothrombotic mechanisms, autonomic inequity favoring sympathetic tone via afferent passageways in the upper airways and lungs, stimulation of the hypothalamic–pituitary–adrenal (HPA) axis by the central nervous system, changes in epigenomic expression, and endothelial barrier dysfunction are just a few of the symptoms. Some of these interconnected mechanisms can respond with high overlap; thus, underlying vulnerability and to detect disease, extra basic risk factors might be needed (e.g., “feed-forward” and reinforce each other). Therefore, it is significant to distinguish (somewhat artificially) the pathways dependent on exposure time courses and the subsequent temporal significance of biological responses. Short-term exposures affect some pathways more than others (e.g., increased risk for thrombosis, autonomic imbalance) and are likely to play a triggering role. Others are expected to take on a long-term role. What are the principal triggering mechanisms of secondary effects, a significant question? Among the three main activating mechanisms direct translocation, oxidative stress, or impacts of elements and subordinate intermediaries produced in response to air contamination are longings that can subsequently arbitrate systemic influences. Notably, neuronal reflexes triggered by the detection of inhaled fragments by distinct receptors in the lungs (possibly without causing oxidative stress) might be regarded as a major effectors’ mechanism. However, they will be considered separately for purposes of clarification.

##### 3.2.7.1. Primary paths of initiation

###### 3.2.7.1.1. PM-mediated consequences of oxidative stress

Oxidative trauma, which can develop in the lung and systemically through several vascular beds, including the blood–brain barrier, may be present in numerous tributary routes (143–146). A more sophisticated model has been replaced by the standard graded response paradigm (147), where ROS and reactive nitrogen species (RNS) interact dynamically, both as site-specific mediators and as core inflammatory regulators (148–150). The first hierarchical response in humans is oxidative stress to air pollution, when additional CV variables are directly evaluated, they show a delayed reaction, indicating that oxidative stress could be an early phase (151). It has been noted that membrane-associated receptors are involved in sensing and transducing particles and particulate components, including initiating inflammatory cascades (152, 153). Several families can activate Toll-like receptors (TLR2/TLR4) along with the nucleotide-binding domain leucine-rich (NOD)-like receptor repeats directly or indirectly may be active, secondary mediators, such as ROS (154–156). Additionally, TRP channels (transient receptor potential) have been implicated, and burning particles or soluble organics can cause oxidative stress (TRPA1 and TRPV1). Depleting antioxidants with low molecular weight will contribute to oxidative stress potential, as can the hereditary predisposition exemplified in antioxidant genes through polymorphisms (145). The chemical half-life of antioxidants and surfactants might be reduced from days to hours and minutes, respectively, by increasing ozone levels from baseline background quantities (30 ppb) to summer smog conditions (100 ppb) (157). It is also predicted that elevated PM_2.5_ and rising ozone concentrations would intensify the effects of PM_2.5_.During phagocytosis, the particle buildup in macrophages might ultimately lead to the initiation of pro-inflammatory pathways, a phenomenon known as frustrated phagocytosis, with long-term exposure (158). Two recent investigations have found that increasing the lung antioxidant barrier via extracellular superoxide dismutase overexpression could reduce the negative complete vascular consequences of air contamination, implying that pulmonary oxidative trauma is important for modulating systemic responses (159, 160).

###### 3.2.7.1.2. Direct translocation

**Figure 2:**
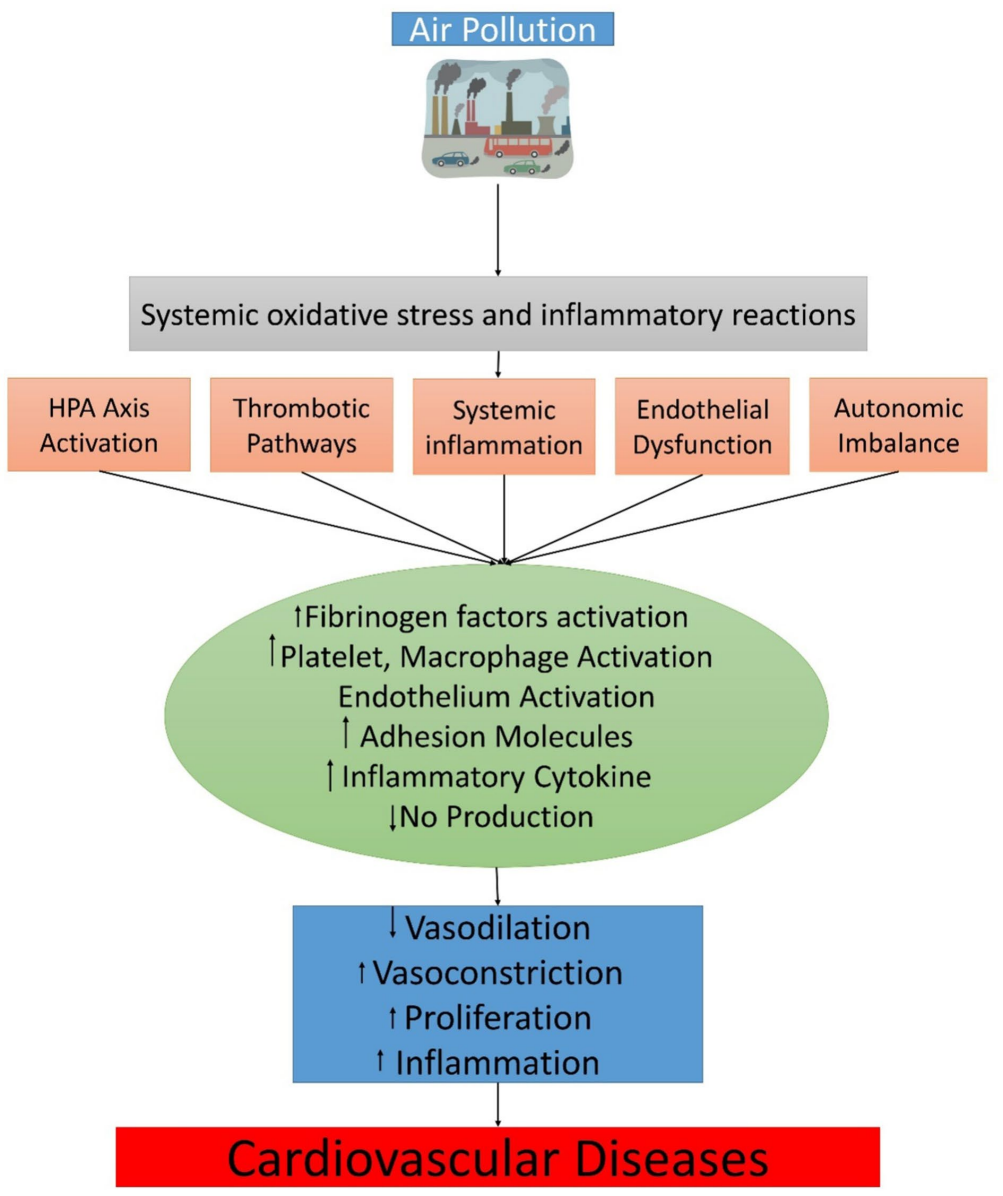
The physiological translational mechanism between air pollution exposure and cardiovascular events.

In specific examples, soluble constituents or other tiny ultrafine elements can be introduced directly into the systemic circulation, resulting in undeviating effects at distant deposition sites (145, 161). In a seminal work in mice and humans, the rapid translocation of inert gold particles into systemic circulation was established (161). Ultrafine particles have been transferred directly in mice across the blood–brain barrier and can be distributed via the production of axonal transport or efferent pathways that control inflammation, blood pressure, and metabolism and are regulated by secondary mediators (Figure 2) (162).

###### 3.2.7.1.3. Biological intermediates as transducers of systemic effects

In addition to exposure, the development of biological intermediates also needs special attention (145, 153, 163–168). Contact with air pollution for a longer time period lowers surfactant resistances (168) and raises oxidative phospholipid-altered substances in surfactant fluids, such as 1-palmitoyl-2-phosphorylcholine (PAPC)-3-arachidonic-sn-glycero and other metabolic byproducts, which may play a role in endothelial barrier dysfunction and the selection of inflammatory cells (Figure 2), facilitating effective signal transfer to the systemic circulation of these and other signals (153). Through activation of TLR4 pathways, these mediators can increase oxidative stress in the vasculature (153). In response to highly concentrated PM_2.5_, impairments in TLR4, NOX2, and neutrophil cytosolic factor 1 (p47^phox^) have mostly been observed to promote vascular function, decrease ROS generation, and decrease inflammatory vasculature monocyte infiltration exposures by inhalation (153, 169). Long-term exposure to PM_2.5_ results in the production of oxidized byproducts like 7-ketocholesterol, which is then transported inside of lipoproteins with low density and subsequently absorbed by CD36, which may be another unusual way that air effluence causes endothelial dysfunction and amplifies the effects of narrowing heart arteries (165, 170). Due to the development of 12-hydroxyeicosatetraenoic acid (12-HETE) and 13-hydroxy octadecadienoic acid (13-HODE), which are peroxidation products of the liver, small intestine, and plasma, exposure to ultrafine particles, like diesel, might exacerbate inflammation and oxidative stress in these areas (171, 172).

###### 3.2.7.1.4. Pathways of secondary effectors

####### 3.2.7.1.4.1. Air pollution causes complete vascular dysfunction and CV restoration

The overwhelming body of research provisions that particle air pollution has rapid and long-lasting effects, in subordinate people and animals on vascular function (3, 75, 111). In animal tests, short, medium, and long-term exposure to air pollution increases superoxide (O_2_) and potentiates vasoconstrictor responses both on its own and in combination with drugs like angiotensin II. At the same time, endothelial function enhances due to the decrease of ROS sources, inflammation, supply of nitric acid, and activation of endothelial cells (adhesion molecule expression). A robust mechanism causing adverse vascular effects may be superoxide production due to uncoupled nitric oxide and nicotinamide adenine dinucleotide phosphate (NADPH) oxidases (173, 174). Inflammatory monocytes had increased microvascular adhesion and peri-vascular deposition of mononuclear cells, with NADPH oxidase (Nox2) and TLR4 deficits enhancing vascular reactivity and swelling with concentrated exposure to PM_2.5_ (153, 175). Even though there have been few type of research comparing ultrafine particles to PM_2.5_, they often demonstrate similar or, in some cases, even more, fantastic impacts, with at least a single research study explaining that contact of mice to ultrafine particles and an apolipoprotein E knockout (ApoE^**−/−**^) resulted in higher atherosclerosis (176). The process could be related to enhancing systemic penetration inflammation, and higher ROS as demonstrated by enhanced LPO products and hepatic malondialdehyde in the liver and plasma, with added pathways that comprise more significant reticence of the anti-inflammatory capability, tremendous systemic oxidative stress, and high-density lipoproteins.

As evidenced by decreased reversible endothelium-dependent or smooth muscle dilation or brief constriction of a reversible peripheral conduit vessel in several monitored human exposure studies, very brief contact with PM_2.5_ along with dilute diesel exhaust causes agonists conduit or microvascular dysfunction. Studies on ultrafine particles (UFPs), including those involving elemental carbon inhalation and watery diesel exhaust, have demonstrated that they can quickly result in endothelial dysfunction in microcirculation (177, 178). Additionally, it was found that exposure to diesel exhaust during exercise caused ST-segment depression and ischemia burden to be significantly higher than filtered air exposure (179). Human data is scarce, which may contribute to the use of higher doses (0.5–1 ppm against the current U.S. National Ambient Air Quality Standard of 0.075 ppm) in animal research; however, ozone exposures in animal studies have been demonstrated to affect endothelial function. It has been observed in human panel reports and in response to concentrated PM_2.5_ disclosure that degradation of endothelial progenitor cells or circulating angiogenic cells can be a significant source of protracted endothelial dysfunction and can also be a significant appliance of sustained endothelial dysfunction. However, the findings are unreliable (160, 180–182). The prevention of lung oxidative stress enhances endothelial progenitor function in the lung by over expressing extracellular superoxide dismutase. Long-term hypertension, diastolic dysfunction, increased cardiac after-load, myocardial fibrosis, alterations in coronary flow reserve, and ultimately left ventricular hypertrophy can result from endothelial failure besides vasoconstrictor’s up-regulation passageways (183). Elevated myosin heavy chain and decreased Sarco/endoplasmic reticulum calcium-ATPase (SERCA2a) suggest aberrant calcium cycling at the molecular level (183).

####### 3.2.7.1.4.2. Autonomic dysfunction other than central nervous system pathway activation

Humans have experienced autonomic nervous system changes that manifest as abrupt changes in sympathovagal equilibrium as seen by variations in plasma stress and heart rate in response to exposure to coarse and small particles. It is now understood that different receptors, including transient receptor potential vanilloid 1 (TRPV1), transient receptor potential ankyrin 1 (TRPA1), and purinergic P2X channels, are stimulated by subtypes of nasal, bronchial, and, pulmonary C-nerve fiber (184–188). These receptors can act as inborn environmental sensors and start the activation of sensory nerves (189). Indeed, blood pressure changes occur in controlled exposure trials in humans in combination with changes in cardiac frequency inconsistency, indicating a sympathetic prevalence at both split ends of the continuum of air contamination. Investigations for a very short time period in canine models and long-term cannulated mice exposed to concentrated air particles showed that hypertension was produced and that there was an indication of central sympathetic activation of the neuron system, presumably induced by neuro-inflammation in relation to the disclosure of PM_2.5_ (190, 191). Ultrafine fragments, nanomaterials, and ozone can both specifically interact with the inducers of circulation factors or the blood–brain barrier in humans and mice and impair neuronal function (162, 168, 192, 193).

####### 3.2.7.1.4.3. Inflammatory reaction at the systemic level

In much early research in animals and people, bone marrow disclosure for a slight time period to air pollution has been demonstrated (3). Recent experimental studies have demonstrated that prolong exposure to concentrated appropriate PM_2.5_ promotes the efflux of Ly6hi + monocytes (CD11b + Gr-1low7/4hi cells) from the bone marrow and, in addition, encourages their subsequent relocation to adipose tissues, the vasculature, and other inflamed tissue, which provides an explanation for the existence of this reaction (75). Since TLR4 deficiency eliminated tissue penetration and ROS production in systemic tissues and reduced the PM_2.5_ influence on boosting peripheral Ly6C^high^ cells (F4/80^+^, CD11b^+^, and CD115^+^), it appears that these pathways play a role in mediating the effects of exposure (153). The strong mobilization of these cells by C-C chemokine receptor type 2 (CCR2) may contribute to insulin resistance/type 2 diabetes in adipose inflammation tissue. CCR2/mice displayed alterations in hepatic lipid aggregation and reduced whole-body insulin tolerance as a result of transcriptional reprogramming mediated by sterol regulatory element-binding protein-1c (SREBP1c) (194). Additionally, chemokine (C-X-C motif) receptor 3 deletion (CXCR3) can be comprised of the translation of PM2.5 influences and transfer populations of T-cells that have been activated (CD44+ CD62L CD4+) (153). In monitored short-term human exposure reports, unequivocal connections between exposure and inflammation have been found less reliably, likely owing to variation in research procedures, participant susceptibility, or previous unmeasured exposures (3).

####### 3.2.7.1.4.4. Prothrombotic pathways

Research has demonstrated on a hamster model of arterial thrombosis, that intratracheal exposure quickly stimulates platelets (195). *Ex vivo* flow chamber perfusion data from human trials showed that breathing inhaled diluted diesel exhaust particulate matter increased thrombotic response and platelet-leukocyte aggregation (196). Direct pulmonary contact or ultrafine PM translocation could cause rapid platelet sensitization. Patients with extra risk factors may be more susceptible to cardiovascular events due to platelet activation and changes in the ratio of tissue plasminogen activator to plasminogen activator inhibitor (179, 197).

####### 3.2.7.1.4.5. HPA-axis activation

Inflammation in white adipose tissue, insulin resistance, and brown adipose dysfunction are all connected to metabolic reprogramming through hypothalamic pathways, according to recent research (75, 133, 134). Metabolic consequences for the short time period associated with insulin resistance frequently appear in human studies. Injecting an inhibitor of the nuclear factor kappa-B kinase subunit (IKK) into the brain prevented the detrimental effects of air pollution on peripheral inflammation, insulin resistance, and whole-body metabolism (191, 198). The quantity of oxidative-modified lipids PAPC that can activate TLR/nuclear factor kappa-B kinase cells (NF-B) in the brain has increased as a result of exposure to PM_2.5_ (198). Activation of the adrenal axis, expressed as elevation of glucocorticoids, can also be an essential mechanism by which air contamination can modulate CV threat (199).

####### 3.2.7.1.4.6. Variations in epigenomics

Environmental influences influence developmental trajectories, according to extensive human and, animal model research and chronic disease vulnerability during crucial prenatal and postnatal growth phases. Environmental epigenetic studies (200) frequently reveal the small epigenetic changes linked with exposure. Although global methylation status has been noted in restricted panel studies, other epigenetic symbols, such as microRNAs, noncoding RNAs, and chromatin alterations, deserve more study now that the technical and financial obstacles to evaluating them are decreasing (201).

## 4. Conclusion

Many strategies for producing toxic effects have been uncovered throughout the last two decades of experimental work on the cardiovascular effects of air pollution and cigarette smoking. Conversely, the specific role of PM and cigarette smoking linked to CVD needs additional elucidation, and this review was intended to encourage exploratory efforts in this capacity. In this review, findings show that cigarette smoke consumption is related to cardiovascular disease; however, the specific elements of cigarette smoke and the appliances underlying this link have not been thoroughly defined in recent quantifiable and tentative research on the potential pathophysiology and mechanisms involved in smoking-related cardiovascular illness. According to epidemiological data, exposure to air pollution raises the risk of myocardial infarction, stroke, and heart failure, which increases both long- and short-term cardiovascular mortality. The inflammatory reaction is an essential part of atherosclerosis development and progression. Several studies have observed that CS induces the peripheral blood leukocyte count to rise by around 20–25%. There have been numerous reviews of the connection between air pollution and hypertension in the past, and at least four recent meta-analyses have focused on this issue. For the following several days, increases in systolic and diastolic blood pressure of 1–3 mm Hg are reliably linked to proliferations in sufficient PM_2.5_ concentrations of 10 g/m^3^. In numerous studies, longer-term exposures have been linked to enduring plasma force increases and an increased prevalence or incidence of hypertension. As a result, interventional studies are necessary to learn more about the direct impacts of cigarette smoking and air pollution on cardiovascular disease.

## 5. Recommendations

### 5.1. Recommendations for cigarette smoking

Based on the available literature, cigarette smoking exposure has been linked to numerous cardiovascular diseases, including coronary artery disease, cardiac arrhythmias, and hypertension. Therefore, the following recommendations can be made:

- Quit smoking: Smoking cessation is the most effective way to reduce the risk of developing cardiovascular diseases associated with smoking. Smokers should be advised and encouraged to quit smoking, and provided with resources and support to assist in the process.
- Avoid exposure to secondhand smoke: Non-smokers should avoid exposure to secondhand smoke as it can also increase the risk of cardiovascular diseases.
- Limit exposure to air pollution: In addition to smoking, exposure to air pollution, particularly particulate matter and gaseous pollutants has been associated with cardiovascular diseases. Therefore, individuals should limit their exposure to air pollution by avoiding outdoor activities during peak pollution hours and using air filters in their homes.
- Exercise regularly: Regular exercise can help reduce the risk of developing cardiovascular diseases associated with smoking. Smokers should be advised to exercise regularly and engage in physical activities that they enjoy.
- Maintain a healthy diet: A healthy diet can also help reduce the risk of developing cardiovascular diseases. Smokers should be advised to consume a diet rich in fruits, vegetables, whole grains, and lean proteins.
- Monitor blood pressure and cholesterol levels: Individuals should have their blood pressure and cholesterol levels checked regularly. If levels are high, they should work with their healthcare provider to manage these conditions through medication and lifestyle changes.

Overall, smoking is a major risk factor for cardiovascular diseases, and individuals should take steps to reduce their exposure to cigarette smoke and other environmental pollutants, as well as adopt healthy lifestyle habits to reduce their risk.

### 5.2. Recommendations for air pollution

Exposure to air pollution has been associated with an increased risk of cardiovascular diseases, including coronary artery disease, cardiac arrhythmias, and hypertension. To reduce the adverse effects of air pollution on cardiovascular health, the following recommendations are suggested:

- Limit exposure to outdoor air pollution: Reduce time spent outdoors during times of high air pollution, particularly during rush hour traffic, wildfires, and industrial activities.
- Use air filters: Install air filters in homes and workplaces to reduce exposure to particulate matter and gaseous pollutants.
- Use public transportation or carpool: Use public transportation, carpool, or bike instead of driving alone to reduce the number of vehicles on the road.
- Quit smoking: Quit smoking or avoid secondhand smoke exposure, as smoking is also a major contributor to air pollution and increases the risk of cardiovascular diseases.
- Promote green energy: Support the development and use of clean and renewable energy sources to reduce reliance on fossil fuels and decrease air pollution.
- Advocate for policy changes: Advocate for stricter regulations and policies to reduce air pollution levels and improve air quality standards.
- Seek medical advice: People with pre-existing cardiovascular diseases should consult with their healthcare providers and follow their advice to minimize their exposure to air pollution and reduce their risk of complications.

Overall, reducing air pollution exposure is crucial to maintaining cardiovascular health, and individuals, policymakers, and healthcare providers should work together to achieve this goal.

### 5.3. Methods

The Study conducted a literature review to examine the links between cigarette smoking, air pollution, and cardiovascular diseases, as well as to summarize the potential mechanisms involved in these associations based on both human and experimental studies. To gather relevant publications, we searched several databases such as PubMed, EBSCO, Cochrane, and Science Direct, using specific MESH terms and keywords such as “cigarette smoking,” “air pollution,” “particulate matter,” “gaseous air pollutants,” “cardiovascular diseases,” and “hypertension.” To ensure the quality of the studies, we applied PECOS criteria that focused on the population (i.e., adults chronically exposed to outdoor air pollutants), comparator (unexposed persons), result (all-cause mortality and cardiovascular diseases), and study design (experimental and epidemiological studies, intervention studies, meta-analyses, and systematic reviews published in peer-reviewed journals in English). We excluded studies involving children, persistent organic compounds, gray literature, conference abstracts, conference papers, comments, editorials, letters, and unpublished data. From the initial 604 papers reviewed, we selected 204 articles for inclusion in our review.

## Author contributions

MM, TS, and MA were responsible for conceptualization, methodology, writing—original draft, visualization. SN, NK, AK, FF, WW, and MN were responsible for writing—review and editing. QZ was responsible for methodology, software, supervision, funding acquisition, writing—review and editing. All authors read and approved the final manuscript.

## Funding

This study was funded by the Natural Science Foundation of Henan Province (Grant 222300420535), China.

## Conflict of interest

The authors declare that the research was conducted in the absence of any commercial or financial relationships that could be construed as a potential conflict of interest.

## Publisher’s note

All claims expressed in this article are solely those of the authors and do not necessarily represent those of their affiliated organizations, or those of the publisher, the editors and the reviewers. Any product that may be evaluated in this article, or claim that may be made by its manufacturer, is not guaranteed or endorsed by the publisher.
